# Editorial: Exploring infectious agents in ruminant foot diseases

**DOI:** 10.3389/fvets.2026.1852474

**Published:** 2026-04-27

**Authors:** Caroline da Silva Silveira, Sergio Fierro, Martin Fraga

**Affiliations:** 1 Plataforma de Investigación en Salud Animal, Instituto Nacional de Investigación Agropecuaria (INIA), La Estanzuela, Colonia, Uruguay; 2 Área de Investigación y Desarrollo, Secretariado Uruguayo de la Lana (SUL), Montevideo, Uruguay

**Keywords:** education, footrot, pododermatitis, risk factor, small ruminant, wild ruminants

Foot diseases remain among the most persistent and impactful health challenges affecting ruminant production systems worldwide. Infections such as footrot, digital dermatitis, contagious ovine digital dermatitis, and other forms of pododermatitis are leading causes of lameness, resulting in substantial economic losses, compromised animal welfare, and reduced productivity across cattle, sheep, goats, and also wildlife populations. These diseases continue to challenge veterinarians, producers, and researchers due to their multifactorial etiology, complex host–pathogen interactions, and strong dependence on environmental and management factors.

The Research Topic *Exploring infectious agents in ruminant foot disease* was conceived to provide an integrated platform for advancing knowledge on infectious foot diseases from complementary scientific perspectives. The Research Topic contributions emphasize that effective progress in this field depends on interdisciplinary approaches that bridge laboratory research, diagnostic pathology, and real-world production systems.

A central theme of this Research Topic is the role of infectious agents. Experimental work in cattle has provided valuable insight into immune responses following infection, recovery, and reinfection with digital dermatitis-associated pathogens (Coatney et al.). Using a controlled induction model, this study demonstrates that although prior exposure may confer partial protection in some animals, classical humoral and cellular immune markers often fail to clearly differentiate protected from non-protected individuals. These findings highlight the complexity of immune responses to digital dermatitis and suggest that protective mechanisms may be localized, transient, or not adequately captured by systemic measurements alone.

Expanding the scope beyond domestic livestock, research on treponeme-associated hoof disease in free-ranging wild elk offers a unique perspective on infectious foot diseases in natural hosts without management intervention (Wilson-Welder et al.). This study shows that immune responses, including antibody titers and lymphocytic proliferation, are more closely associated with disease severity than with protection. Notably, animals with advanced lesions exhibited stronger immune responses, while clinically healthy individuals showed little evidence of naturally acquired immunity. These findings reinforce the hypothesis that naturally occurring protective immunity against treponeme-driven hoof diseases may be rare, underscoring the challenges faced in vaccine development and long-term disease control.

Pathology-based investigations included in this Research Topic further strengthen the diagnostic foundation of infectious foot diseases. By integrating clinical evaluation with histopathological and radiographic analyses, a refined classification system for pododermatitis in sheep has been proposed (da Silva Silveira et al.). This work demonstrates that foot lesions are progressive and frequently involve deeper tissues and bone structures as severity increases, regardless of the initiating infectious agent. Such integrative diagnostic approaches provide essential tools for improving prognostic accuracy, guiding treatment decisions, and supporting evidence-based management strategies in the field.

In addition to biological and pathological insights, this Research Topic acknowledges the importance of human behavior, perception, and education in the epidemiology and control of infectious foot diseases. A survey of sheep producers in Australia identified key risk factors for footrot and other hoof diseases, including flock size, biosecurity practices, inspection frequency, and farm topography (Smith et al.). While most producers recognized the negative impacts of foot diseases on productivity and welfare, the study highlights how practical constraints and management decisions shape disease outcomes, emphasizing the need for context-specific control strategies.

Complementing the producer perspective, research exploring veterinary education revealed important gaps in undergraduate training related to lameness in sheep (Clifton et al.). Limited teaching time and restricted clinical exposure were associated with low confidence among students in diagnosing and managing foot diseases. These findings highlight that scientific advances alone are insufficient unless accompanied by effective knowledge transfer to future veterinarians and, ultimately, to producers.

Collectively, the studies presented in *Exploring Infectious Agents in Ruminant Foot Disease* illustrate that infectious foot diseases are dynamic, multifaceted problems that require integrated solutions. Advances in immunology, pathology, and epidemiology must be aligned with education, extension, and on-farm realities to achieve meaningful and sustainable improvements in animal health and welfare.

By bringing together research across species, disciplines, and production contexts, this Research Topic provides a comprehensive overview of current challenges and emerging insights in the field of ruminant foot diseases. It is our hope that these contributions will stimulate further interdisciplinary collaboration, inform the development of improved diagnostic and control strategies, and ultimately contribute to reducing the global burden of lameness in ruminant populations.

